# A simple questionnaire to prevent blindness: a cross-sectional study screening for Stickler syndrome in children

**DOI:** 10.1093/rheumatology/keag389

**Published:** 2026-07-31

**Authors:** Robert Smyth, Peter Bale, Thomas R W Nixon, Annie M McNinch, Howard Martin, Allan J Richards, Martin P Snead

**Affiliations:** Paediatric Rheumatology Department, Cambridge University Hospitals, Cambridge, UK; Department of Clinical Neurosciences, University of Cambridge, Cambridge, UK; Vitreoretinal Research Group, John van Geest Centre for Brain Repair, University of Cambridge, Cambridge, UK; NHS England Stickler Syndrome Highly Specialised Service, Cambridge University Hospitals NHS Trust, Cambridge, UK; Paediatric Rheumatology Department, Cambridge University Hospitals, Cambridge, UK; NHS England Stickler Syndrome Highly Specialised Service, Cambridge University Hospitals NHS Trust, Cambridge, UK; Vitreoretinal Research Group, John van Geest Centre for Brain Repair, University of Cambridge, Cambridge, UK; NHS England Stickler Syndrome Highly Specialised Service, Cambridge University Hospitals NHS Trust, Cambridge, UK; Vitreoretinal Service, Cambridge University Hospitals NHS Foundation Trust, Cambridge, UK; Vitreoretinal Research Group, John van Geest Centre for Brain Repair, University of Cambridge, Cambridge, UK; NHS England Stickler Syndrome Highly Specialised Service, Cambridge University Hospitals NHS Trust, Cambridge, UK; Vitreoretinal Research Group, John van Geest Centre for Brain Repair, University of Cambridge, Cambridge, UK; NHS England Stickler Syndrome Highly Specialised Service, Cambridge University Hospitals NHS Trust, Cambridge, UK; Vitreoretinal Research Group, John van Geest Centre for Brain Repair, University of Cambridge, Cambridge, UK; Department of Clinical Neurosciences, University of Cambridge, Cambridge, UK; Vitreoretinal Research Group, John van Geest Centre for Brain Repair, University of Cambridge, Cambridge, UK; NHS England Stickler Syndrome Highly Specialised Service, Cambridge University Hospitals NHS Trust, Cambridge, UK; Vitreoretinal Service, Cambridge University Hospitals NHS Foundation Trust, Cambridge, UK

**Keywords:** Stickler syndrome, *COL2A1,* retinal detachment, skeletal dysplasia, myopia, cleft palate

## Abstract

**Objectives:**

Stickler syndrome is a skeletal dysplasia that is widely under-recognized yet the most common cause of inherited retinal detachment in children. The most prevalent subtype is Stickler syndrome type 1 (STL1), with an autosomal dominant variant in *COL2A1* typically resulting in ophthalmic, orofacial, auditory and musculoskeletal manifestations. However, manifestations across these domains are not as prominent in children, making identification of individuals suitable for further assessment difficult. Thus, we sought to test an easy-to-use screening tool capable of differentiating children with STL1 from the general paediatric population.

**Methods:**

Children aged 4–10 years with and without *COL2A1*-confirmed STL1 were recruited from a single specialist centre and online via the national patient support group Stickler Syndrome UK (Registered Charity number 1060421). A screening tool was administered, and planned analysis of the median final score and individual components of the questionnaire was completed.

**Results:**

Twenty-six general paediatric and twenty-eight STL1 participants were recruited. STL1 participants scored higher on screening tool final score with a median score (IQR) of 7 (3.5) *vs* 0 [1], which was statistically significant on a Mann–Whitney *U* test (*P* < 0.001). The sensitivity and specificity were 93% and 96%, respectively, and STL1 participants were more likely to have myopia, palate abnormalities, family history of cleft palate and hypermobility on secondary analysis of the individual screening tool components (Hochberg-corrected all *P* < 0.005).

**Conclusion:**

We demonstrate a clinically powerful screening tool capable of identifying children with underlying STL1, and thus this questionnaire can now be applied to at-risk children to inform the need for further specialist assessment.

Rheumatology key messages

- Stickler syndrome is the most common cause of inherited retinal detachment in children, but early identification can prevent blindness.
- Our easy-to-use questionnaire differentiates *COL2A1*-associated Stickler syndrome Type 1 paediatric patients from the general population.
- This questionnaire can be used as a screening tool in at-risk populations, including children with myopia, cleft palate, hypermobility and/or retinal detachment to help guide referrals for specialist ophthalmic review.

## Introduction

The Stickler syndromes are a group of inherited skeletal dysplasias affecting type II, IX or XI collagen synthesis [1]. They are a widely under-recognized group of conditions, yet are the most common cause of familial retinal detachment and particularly retinal detachment and vision loss in children [2]. The NHS England Highly Specialised Service for Stickler syndrome at Cambridge University Hospitals NHS Trust (CUH) is the only such service in the UK and Ireland with over 2000 registered patients, but it is likely there are many more undiagnosed cases across the UK and Ireland, as well as worldwide.

The most common variety of Stickler syndrome, accounting for around 80% of patients, is Type 1 (STL1), caused by an autosomal dominant variant in the gene for type II collagen, *COL2A1* [1]. STL1 exists at the milder end of the *COL2A1*-associated skeletal dysplasias, and typically presents across four domains, with musculoskeletal (hypermobility, joint pain and early-onset arthritis, spinal malformations, skeletal dysplasia, Perthes disease), ophthalmic (myopia, retinal detachment, pathognomonic congenital vitreous changes), orofacial (cleft palate, Pierre Robin sequence, bifid uvula or high-arched palate, midfacial hypoplasia) and auditory (mild high-pitch sensorineural hearing loss, or conductive hearing loss in conjunction with cleft palate and eustachian tube dysfunction) manifestations [2–8]. Retinal detachment is perhaps the most important of these, with up to 70% of patients with STL1 likely to experience it in their lifetime [1]. Crucially, presentation is often delayed in the paediatric population, where children may not report vision loss in one eye, or not until there is involvement of the second eye. This all contributes to up to 30% of Stickler patients suffering avoidable and lifelong vision loss in at least one eye [3, 9].

Prophylactic treatment is highly effective in reducing the risk of visual loss from retinal detachment, provided patients are identified in a timely manner before retinal detachment occurs. It has clearly been shown in STL1 patients to decrease the risk of future detachment by 5–10-fold [9–14]. Administering this treatment throughout the population prevents avoidable sight loss and blindness in children. New methods to address underdiagnosis are urgently required to allow access to this intervention.

One potential solution is a simple screening tool for Stickler syndrome that could be easily deployed in a general clinic setting. This could be applied to at-risk populations by any clinician, identifying patients who may warrant further assessment and/or genetic testing for Stickler syndrome. Wang *et al.* proposed one such screening tool to be used in the Perthes disease population [15]. They observed a case series of STL1 patients with historic diagnoses of Legg-Calve-Perthes disease, an avascular necrosis of the hip that typically presents between 4–10 years of age, prior to the median onset of retinal detachment in STL1 [16]. They proposed a questionnaire that collected information in the four domains of Stickler syndrome, and a scoring system to assign patients without a previous STL1 diagnosis to a high- or low-risk category. By applying this to an at-risk population, here the paediatric Perthes disease population, those with underlying STL1 could be identified. Of the 10 patients diagnosed with Perthes disease before retinal detachment in Wang *et al.* [15]’s case series, a high-risk category would have been assigned to nine of them. This would have allowed the provision of prophylactic cryotherapy and prompted further screening of families due to the autosomal dominant inheritance pattern of retinal detachment, preventing avoidable sight loss. In particular, one case (patient 14) lost sight in both eyes at 19–20 years due to retinal detachment, having previously presented with Perthes disease at 5 years. With congenital high myopia and a family history of retinal detachment, this is a clear example where blindness could have been prevented if STL1 was identified by a screening tool.

However, this proposed screening tool has not been tested in an independent paediatric population, and thus there is currently no evidence it can distinguish STL1 children from the general population with sufficient sensitivity and specificity. Thus, we report a cross-sectional study of paediatric STL1 and control patients who have been assessed using a screening tool in order to test whether family and medical history can be used to identify paediatric patients with a high risk of STL1.

## Methods

### Ethical approval

Prior to research commencement, this study received Research Ethics Committee and Health Research Authority approval via IRAS [Integrated Research Application System (IRAS) number 343181]. The study protocol was also registered on the UK’s Clinical Study Registry [International Standard Randomised Controlled Trial Number (ISRCTN) 86229394]. The research proposal was presented at the national conference 2024 of the patient support group Stickler Syndrome UK (SSUK, Registered Charity number 1060421), and SSUK were updated on the progress and results of the study throughout. National Institute for Health and Care Research Good Clinical Practice training was completed by all researchers involved in data collection and handling. All participants and their parents received appropriate participant information sheets and gave full informed consent to participate in the study, and procedures followed were in accordance with the ethical standards of the Helsinki Declaration.

### Screening tool inclusions and scoring

The screening tool from Wang *et al.* was reviewed based on the available literature, observations of experienced clinicians at CUH, and data collected from Wang *et al.*’s Stickler-Perthes case series [1–8, 15]. The majority of the proposed screening tool was retained. Two small caveats were added: that ‘high-arched palate/bifid uvula’ would only be assessed if there had been no previous cleft palate repair and that a Beighton score for hypermobility of ≥6 would be considered positive, in line with most recent paediatric advice [17]. Short stature was removed as it is not seen consistently across the population when distinguished from spondyloepiphyseal dysplasia congenita [3, 7, 18]. Furthermore, a family history of atraumatic total hip replacement was added to reflect the significant joint pain from early-onset arthritis experienced in the Stickler population [19]. Whilst other considerations included midface hypoplasia, genu valgum and valvular cardiac disease, these were not included due to difficulty in defining or insufficient evidence in Stickler syndrome [2, 7, 20–22]. Perthes disease was also excluded, with the risk of biasing the tool towards Wang *et al.* [15]’s previous Perthes population, and elbow hyperextension alone as an alternative for Beighton scoring was not implemented, as it could be assessed alongside Beighton scoring in the secondary analyses [7]. The study screening tool can be seen in Table 1.

**Table 1:** Proposed screening tool, adapted from Wang *et al*. [15].

Ophthalmic	Myopia (short-sightedness) before the age of 10 years	2
	Retinal detachment	4
	Family history of retinal detachment: single relative	2
	Family history of retinal detachment: >1 relative	4
Orofacial	History of cleft palate repair	3
	High-arched palate or bifid uvula on examination	2
	Family history of cleft palate repair	2
Auditory	Sensorineural hearing loss	2
	Family history of deafness	1
Musculoskeletal	Spinal abnormalities (flattened vertebrae, end plate changes, scoliosis, kyphosis)	2
	Family history of total hip replacement aged <50 years	2
	Hypermobility on examination (Beighton score ≥6)	1

Scores for each positive item are recorded on the right-hand column. Questionnaire items were ordered by domain on the left, but this did not contribute to scoring.

### Participant recruitment

A population of patients aged 4–10 years with and without Stickler syndrome were selected from single specialist centre (CUH). Participants with Stickler syndrome were recruited from patients attending the Stickler clinic in CUH between 1 May and 14 August 2025, aged between 4 and 10 years with a confirmed pathogenic *COL2A1* variant. Participants were also recruited via the SSUK patient membership survey. This includes Stickler syndrome patients and their families located across the UK, Ireland and beyond with an interest in participating in research. Emails were sent requesting participants who fulfilled the above criteria to complete the SSUK eSurvey via a confidential NHS-linked Microsoft form.

A control population of general paediatric patients without Stickler syndrome in the same age range were recruited. These were selected from general paediatric clinic attendees in CUH.

### Exclusions

Any participants with another genetically confirmed condition, formally diagnosed connective tissue disorder, or previous diagnosis of Perthes disease were excluded.

### Completion of screening tool

All participants recruited at CUH were asked each questionnaire item in turn, marking ‘yes’ or ‘no’ to each. Any clarifications were noted at the time of asking. One researcher (R.S.) administered all in-person scores to reduce inter-assessor variability. All participants consented to review of their electronic patient record to confirm the inclusion and exclusion criteria, and items of the screening tool. As it was not possible to blind participants to their diagnosis, the items in the screening tool were worded to be as objective as possible.

Participants from SSUK completing the questionnaire electronically were asked to provide an answer of ‘yes’, ‘no’ or ‘unsure’, to each questionnaire item in turn. Self-administered Beighton scoring was requested where participants were unsure about extent of hypermobility.

### Statistical analysis

All analysis was completed using RStudio 2025.05.1 + 513. The primary analysis was a two-tailed Mann–Whitney *U* test on the final screening tool scores of the Stickler and control groups, with a null hypothesis of no difference. At least 26 patients were required per group as calculated by G*power 3.1, assuming large effect size (d = 0.8), α error of 5% and power of 80% [23]. The sensitivity and specificity of the tool was also calculated as part of the primary analysis, with a cut-off score of ≥4 used to classify participants as ‘high-risk’. A receiver operating characteristic (ROC) curve was plotted to compare different cut-offs.

Secondary analysis included the individual elements of the screening tool, with their Hochberg-corrected significance calculated using Chi-squared tests, Fisher’s exact test or goodness-of-fit testing. The individual elements of the Beighton score were analysed in a similar manner. The final score was also assessed against age by group-wise linear regression analysis. Age, sex, ethnicity and location data were also analysed.

Any missing data was excluded from the final statistical analysis but recorded in the appropriate results section and supplementary data.

## Results

### Participants

Twenty-eight participants with STL1 and twenty-six paediatric control participants aged between 4 and 10 years were recruited. Twenty-three of the Stickler participants were recruited from the CUH Stickler clinic, whilst five were recruited from the SSUK eSurvey. All participants with STL1 had confirmed pathogenic *COL2A1* variants (Table 2). All participants completed consent forms and questionnaire in full and met inclusion/exclusion criteria. No participants were excluded during analysis, and one participant who had turned 11 years old during the recruitment period was allowed to continue with the questionnaire. Full anonymised individual screening tool results and genetic variants are detailed in Supplementary Table S1.

**Table 2:** COL2A1 variants in the recruited Stickler participants, alongside their age, final screening tool score and the variant exon and effect.

Participant ID	Age (years)	Final score	COL2A1 variant (NM_001844.5)	Exon	Variant effect
S1	8	6	c.2T > C p.?	1	Alteration to initiation codon
S2	4	7	c.491dupC p. Gly165TrpfsTer24	7	Frameshift
S3	8	9	c.654 + 4_654 + 7delAGTA	9	Missplicing
S4	9	5	c.659_661delCTC p. Pro220del	10	In-frame deletion
S5	7	2	c.701G > T p. Gly234Val	10	Missense
S6	4	8	c.1222-1G > C	20	Missplicing
S7	4	6	c.1366-1G > A	22	Missplicing
S8	9	5	c.1420-9G > A	23	Missplicing
S9	4	4	c.1527 + 135G > A	23	Missplicing
S10	7	9	c.1693C > T p. Arg565Cys	26	Missense
S11	8	8	c.1996-9G > A	31	Missplicing
S12	9	7	c.2050-1G > A	32	Missplicing
S13	11	7	c.2101C > T p. Arg701Ter	33	Nonsense
S14	4	7	c.2195delG p. Gly732ValfsTer56	34	Frameshift
S15	10	3	c.2659C > T p. Arg887Ter	40	Missense
S16	4	4	c.2703delT p. Ala902LeufsTer126	41	Frameshift
S17	5	7	c.2710C > T p. Arg904Cys	41	Missense
S18	6	4	c.2710C > T p. Arg904Cys	41	Missense
S19	8	4	c.2794C > T p. Arg932Ter	42	Nonsense
S20	6	12	c.2976_2977delAG p. Gly993IlefsTer10	43	Frameshift
S21	8	12	c.3106C > T p. Arg1036Ter	44	Nonsense
S22	5	11	c.3137delC p. Pro1046LeufsTer84	45	Frameshift
S23	10	4	c.3165 + 1G > T	45	Missplicing
S24	6	5	c.3261_3262delCA p. Asp1087GlufsTer30	46	Frameshift
S25	8	8	c.3574C > T p. Arg1192Ter	50	Nonsense
S26	5	7	c.3574C > T p. Arg1192Ter	50	Nonsense
S27	10	9	c.3597 + 2T > G	50	Missplicing
S28	6	9	c.4011G > A p. Trp1337Ter	52	Nonsense

Genetic variants are expressed in HGVS nomenclature with the copy number and affected amino acid where appropriate. Copy number variants with a ± show variants present in the intron sequence and are associated with missplicing. The affected/adjacent exon and general description of the resulting variant effect is listed. The matched participant’s final screening tool score was also displayed.

### Demographics

There was no difference in median age (7 years in both) or sex between the Stickler and control groups. Full demographic data can be found in Supplementary Data S1.

### Primary outcome: final screening tool score

The primary outcome measure was the median final score of the screening tool in each group. The median screening tool score in paediatric patients with STL1 was 7 (IQR 3.5, range 2–12), whilst in the control group, it was 0 (IQR 1, range 0–6). As the data for the control group was not normally distributed, a two-tailed Mann–Whitney *U* test was performed, which showed a significant difference between the two groups (*P* < 0.001).

**Figure 1:**
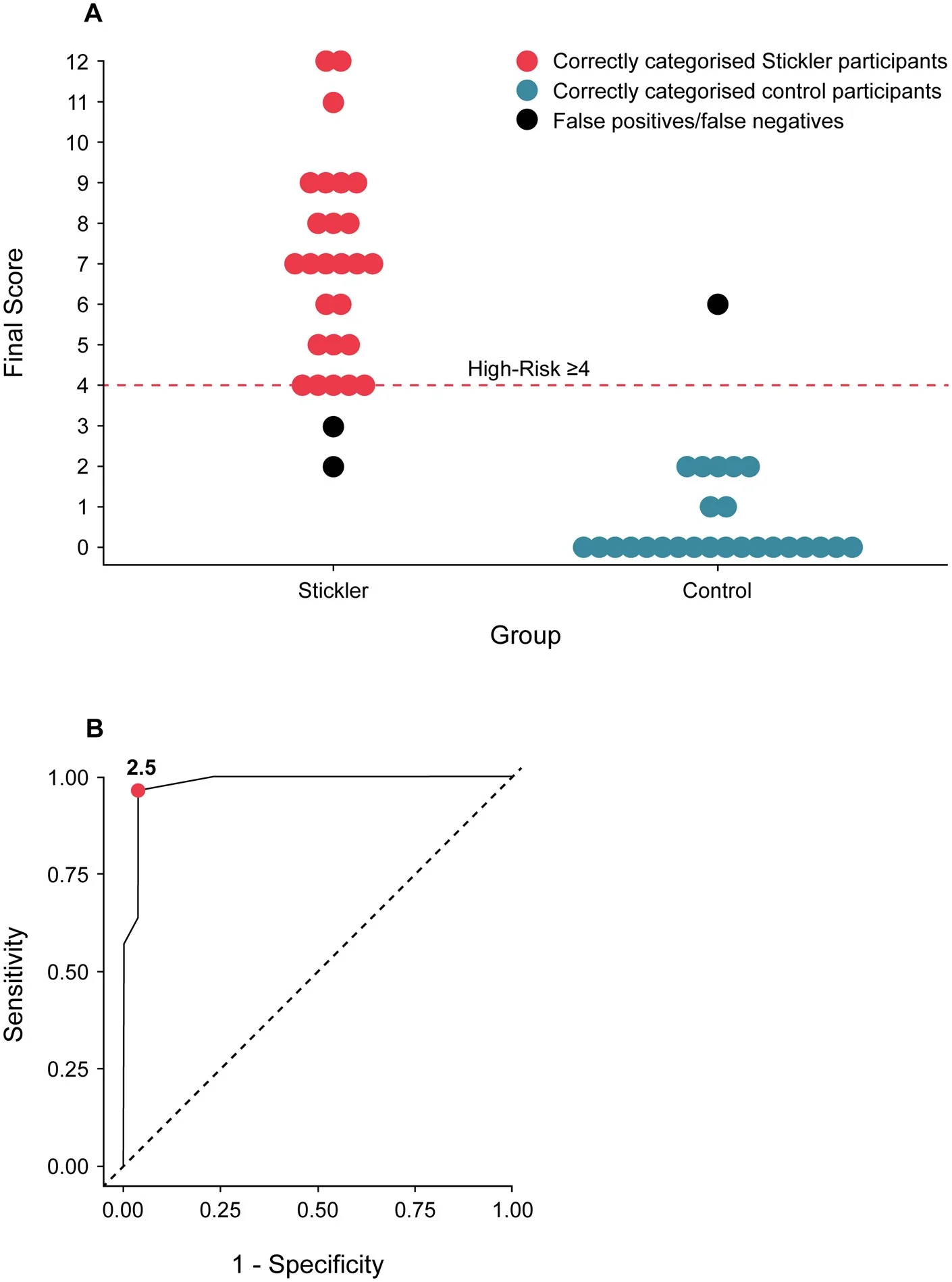
Jitter plot of final scores for Stickler and control groups with cut-off and ROC curve for cut-offs. (**A**) Jitter plot of final score against Stickler and control groups with cut-off. The cut-off score is illustrated by the red dotted line, with scores ≥4 considered ‘high-risk’. Correctly categorized Stickler participants are represented by red dots, whilst correctly categorized control participants are blue. Black dots represent the false-negative and false-positive final scores of participants in both groups. (**B**) ROC curve for screening tool final score sensitivity and specificity using different thresholds. The optimal threshold score of 2.5 (i.e. ≥3) is labelled, with a Youden’s index J score of 0.926 (sensitivity 96%, specificity 96%). The *a priori* threshold of ≥4 has the second highest Youden’s index J score of 0.890 (sensitivity 93%, specificity 96%)

A cut-off of ≥4 had been proposed as a classification for ‘high-risk’ Stickler participants. Using this, 26 of 28 Stickler participants were classed as high-risk, whilst only 1 of 26 general paediatric participants were classed as high-risk (Fig. 1A). This gives an overall sensitivity of 93% and specificity of 96% for the screening tool. An ROC curve was also created, with an optimal threshold of 2.5 (i.e. ≥3) calculated using Youden’s index (Fig. 1B).

### Secondary outcomes: individual screening tool components

On secondary analysis, the following individual components of the screening tool appeared at a significantly different frequency in the Stickler *vs* control groups: myopia, palate abnormalities, family history of cleft palate and hypermobility (Hochberg-corrected *P* < 0.001, *P* = 0.004, *P* = 0.005, *P* < 0.001, respectively). Full analysis is detailed in Table 3, below. Retinal detachment was not significantly different between the Stickler and control groups, with the only retinal detachment recorded being a participant from the SSUK eSurvey originating in the US. However, of the Stickler participants, 25 of 28 (89%) had received prophylactic cryoretinopexy treatment to prevent retinal detachments.

**Table 3:** Summary of frequencies of individual screening tool components per group.

Questionnaire item	Stickler Y/N (%) (N = 28)	Control Y/N (%) (N = 26)	*P*-value	Adjusted *P*-value (Hochberg)
**Ophthalmic**				
Myopia	26/2 (93%)	3/23 (12%)	<0.001	<0.001*
Retinal detachment	1/27 (4%)	0/26 (0%)	1.000	1.000
Family history of retinal detachment	10/18 (36%)	3/23 (12%)	0.063	0.317
In >1 relative	4 (14%)	0 (0%)	—	—
In 1 relative	6 (21%)	3 (12%)	—	—
None	18 (64%)	23 (88%)	—	—
**Orofacial**				
Palate abnormalities	19/6 (76%)	1/25 (4%)	<0.001	0.004*
Cleft palate	13 (52%)	0 (0%)	—	—
High-arched palate or bifid uvula	6 (24%)	1 (4%)	—	—
None	6 (24%)	25 (96%)	—	—
Family history of cleft palate	10/17 (37%)	0/26 (0%)	<0.001	0.005*
**Auditory**				
Sensorineural hearing loss	5/23 (18%)	0/26 (0%)	0.052	0.311
Family history of deafness	2/26 (7%)	2/24 (8%)	1.000	1.000
**Musculoskeletal**				
Spinal abnormalities	2/26 (7%)	0/26 (0%)	0.491	1.000
Family history of total hip replacement	1/27 (4%)	1/25 (4%)	1.000	1.000
Hypermobility	16/12 (57%)	0/26 (0%)	<0.001	<0.001*

An appropriate statistical test was applied to each question: a Chi-squared test (2 × 2 groups with >5 expected responses per group; myopia and hypermobility), Fisher’s exact test (2 × 2 groups with ≤5 expected responses per group; retinal detachment, family history of cleft palate, sensorineural hearing loss, family history of deafness, spinal abnormalities and family history of total hip replacement) or Goodness of Fit test (2 × 3 groups; family history of retinal detachment and palate abnormalities). Hochberg’s correction for multiple *P*-values was applied to give a final value and conservative indication of significance, as indicated by asterisks. There were four missing data points from SSUK eSurvey participants: three missing entries where participants and their families were unsure of the presence of a high-arched palate or bifid uvula, and one missing entry where participants and their families were unsure of the presence of a family history of cleft palate. One of the SSUK participants was able to score their hypermobility using a Beighton score. One Stickler participant and one control participant reported no high-arched palate or bifid uvula but were found on examination to have a high-arched palate and bifid uvula respectively.

### Secondary outcomes: individual hypermobility Beighton score components

Hypermobility was calculated using the Beighton score [24]. The median score for the Stickler group was 6.00 (IQR 4.50–7.00) *vs* 2.00 (IQR 0.50–3.00) for the control group. This was significantly different using a Mann–Whitney two-sided *U* test (*P* < 0.001). A participant was classed as positive for hypermobility if they scored ≥6 on the Beighton score on examination, with 16 of the 28 in the Stickler group positive and none of the control group. The specific joints being scored were recorded in 17 of the 28 Stickler participants and 18 of the 26 control participants (Table 4). Elbow and finger hyperextension were significantly different between the Stickler and control groups, whilst right knee hyperextension was just significant once Hochberg correction was applied. None of spine, left knee or thumb hyperextension were significantly different between the two groups. Notably there were missing Beighton scores for 19 participants, including four of the five SSUK-recruited participants, two of whom self-reported as hypermobile without a specific score.

**Table 4:** Summary of frequencies of individual Beighton score components per group.

Beighton score item	Stickler Y/N (N = 17)	Control Y/N (N = 18)	*P*-value	Adjusted *P*-value (Hochberg)
Elbow (Left)	17/0	7/11	<0.001	0.001*
Elbow (Right)	17/0	7/11	<0.001	0.001*
Finger (Left)	10/7	0/18	<0.001	0.001*
Finger (Right)	11/6	0/18	<0.001	<0.001*
Thumb (Left)	11/6	7/11	0.127	0.253
Thumb (Right)	11/6	6/12	0.063	0.190
Knee (Left)	9/8	3/15	0.024	0.095
Knee (Right)	9/8	2/16	0.008	0.039*
Spine	3/14	5/13	0.691	0.691
**Hypermobility**	16/12 (57%)	0/26 (0%)	<0.001	<0.001*
**Beighton score (median (IQR))**	6.00 (4.50–7.00)	2.00 (0.50–3.00)	Mann–Whitney *U* = 34, *P* < 0.001*	

An appropriate statistical test was applied: Chi-squared test (2 × 2 groups with >5 expected responses per group; left and right elbow, right finger, left and right thumb, left and right knee, and hypermobility), and Fisher’s exact test (2 × 2 groups with ≤5 expected responses per group; left finger and spine). Hochberg’s correction for multiple *P*-values was applied to give a final value and conservative indication of significance, as indicated by asterisks. The screening tool hypermobility score in both groups from Table 3 was included for context. A two-tailed Mann–Whitney *U* test was also applied to the median Beighton scores for each group, showing a significant difference. There were 19 missing entries where full Beighton scores were not recorded, with 11 missing from the Stickler group, and eight from the control group. One SSUK eSurvey participant who was unsure of the presence of hypermobility was able to self-assess as hypermobile via an electronic copy of the Beighton score.

### Final screening tool score by age

Group-wise linear regression analysis showed no relationship between screening tool final score and age in the Stickler or control groups (Supplementary Fig. S1).

## Discussion

This research set out to test the hypothesis that paediatric patients with a high risk for Stickler syndrome could be identified by an easy-to-use screening tool without specialist ophthalmic assessment. There was a clear difference in the final score between those with and without Stickler syndrome, and the proposed cut-off score of ≥4 resulted in a high sensitivity (93%) and specificity (96%). There was also no trend between screening tool final score and age, implying that the tool may remain reliable for distinguishing Stickler syndrome in children younger than 4 years and older than 10 years.

This screening tool has some clear advantages over previously ratified diagnostic tools. These tended to focus on the presence of multiple manifestations across the four domains of STL1, meaning that children, who have not had time to develop these manifestations, are especially at risk of suffering retinal detachment before diagnosis [4, 18, 25]. Furthermore, the pathognomonic vitreous changes of STL1 included in diagnostic tools require specialist ophthalmic examination, which may either be unavailable or difficult in a young child, further precluding diagnosis for example in paediatric rheumatology/musculoskeletal clinics or cleft palate services where these children often first present. Additionally, STL1 presentations can be subtle, meaning not all criteria of a diagnostic tool will be obviously met. For example, the typically described midface hypoplasia will not be prominent in many patients and ethnic groups, and thus a compounding lack of clinician awareness/experience in STL1 will result in these patients not being referred for specialist assessment [20, 21].

This screening tool alleviates these concerns and gives healthcare professionals the ability to confidently identify paediatric patients at risk of STL1. Retinal detachment is typically described in the second to third decade of life, but can occur earlier, and the ability to refer children with suspected STL1 in the first decade of life prevents sight loss.

However, despite the high sensitivity and specificity, some cases were mislabelled. The one false-positive case had mild myopia (−0.25D), retinal detachment in maternal grandmother and a family history of hip replacement related to developmental dysplasia of the hip, resulting in their score of 6. This tool is a screening tool, with further assessment recommended in all ‘high-risk’ patients, meaning a small number of false positives that do not match a true Stickler phenotype is necessary to ensure cases are not missed.

False negative cases are more concerning. Of the two false-negative cases, one had myopia only, and the second had myopia alongside hypermobility. Both these missed STL1 cases represent *de novo* mutations, where family history is not present. De novo participants had a lower median final score (IQR, number of participants) of 5.5 (3.0, 12) than familial variant participants with 7.5 (3.25, 16). This was statistically significant in post-hoc analysis without correction (Mann–Whitney *U* test = 50.5, *P* = 0.035). Though this result should be interpreted cautiously, it suggests there may be a risk of missing these *de novo* cases. This is particularly pertinent for those with ocular-only Stickler syndrome, where a COL2A1 variant in exon 2 is spliced out of mature cartilage tissues other than the vitreous membrane of the eye, making presentation to ophthalmology teams alone likely [26, 27]. Our calculated Youden’s index recommended lowering the ‘high-risk’ cut-off score from ≥4 to ≥3 and implementing this would eliminate one of these false negatives. However, children presenting with myopia only in STL1 remain at high risk of undetected retinal detachment.

Of the individual components of the screening tool, Stickler participants were more likely to display myopia, palatal abnormalities, family history of cleft palate and hypermobility. This generally matches the common pathways for referral [2]. Hypermobility, however, was surprisingly specific. Previous studies have described high rates of hypermobility amongst the general paediatric population, but we have found good specificity when using a higher Beighton score (≥6 *vs* ≥ 4), in line with most recent evidence and recommendations [17, 28, 29]. Indeed, given the high specificity with the current Beighton scoring method, it would be reasonable to increase the score of this component from 1 to 2.

Prior to the study we had considered using elbow hyperextension alone as a marker of hypermobility. We found it present bilaterally in all STL1 participants who had fully recorded Beighton scores and thus suggest that in the absence of a full Beighton score, elbow hyperextension is a sensitive alternative. Indeed, we anecdotally noted that elbow hyperextension was exaggerated and obvious in these children with Stickler syndrome in line with previous studies [7]. However, the specificity is low, with 39% of the control group also displaying some degree of elbow hyperextension. If used instead of the full Beighton score, it should only merit a score of 1. These results should be considered conservatively given the number of patients without a recorded Beighton score. It is also important to note that a degree of hypermobility in the paediatric population is normal despite being an over-reported clinical concern. In our experience, it is also often poorly self-scored. Thus, in this screening tool, hypermobility alone is not an indicator of STL1, and it is expected to present with other manifestations as above.

There was no difference in retinal detachment between Stickler and control groups; the sole case was an SSUK eSurvey participant living in the USA without access to CUH’s prophylactic cryoretinopexy. With 89% (25/28) of the participants having received prophylaxis prior to the study, the low rates of retinal detachment support the previous well-established studies that the risk of retinal detachment is markedly reduced by prophylaxis [9–14]. Importantly, it also shows that this screening tool can identify patients before retinal detachment occurs.

In the auditory domain, relatively few patients had symptoms identified. Hearing loss in STL1 is subtle compared with COL11, COL9 or other genes associated with Stickler syndrome, with mild high pitch sensorineural hearing loss generally reported [8, 30]. Indeed, conductive or mixed hearing loss is as common in STL1. It is possible that this domain will prove more beneficial when applied to patients with non-COL2A1 Stickler syndrome.

In the musculoskeletal domain, only hypermobility showed significant differences. Spinal abnormalities are well described in Stickler syndrome, but routine spinal X-rays are not recommended without clinical signs and thus patients may be unaware of radiological changes. Both participants with spinal abnormalities here reported scoliosis. Family history of total hip replacement also did not distinguish between groups, with the one instance in the control group already described above as part of the false-positive case. Though hip replacements at a young age associated with trauma are easy to filter out, ruling out other conditions such as developmental dysplasia of the hip is more difficult in a non-expert screening tool. This item will remain as part of the screening tool due to its potential in identifying familial Perthes disease, which is COL2A1-associated, and the preference for false positives over negatives [15].

There were some limitations to this study. It was not practical to blind participants or assessors in our study to the underlying diagnosis of STL1. This does mean families with formally diagnosed STL1 are more likely to recall relevant family history, and an assessor is more likely to mark subjective measures, such as high-arched palate (where there is no formal clinical measure), as positive. This was minimized by not calculating final scores until after data collection was completed and using objective diagnoses and family histories as far as possible. Inter-assessor variability was also reduced by using the same assessor for in-person screening.

A separate limitation of this research is that the screening tool is primed towards STL1. This is because of the availability of prophylaxis for patients with STL1, thus fulfilling one of Wilson’s criteria for a screening tool. However, almost 20% of patients with Stickler syndrome will have one of the other types, and early diagnosis to manage ophthalmic, orofacial, auditory and musculoskeletal manifestations remains important. Whilst many of the inclusions on this screening tool are consistent with other types of Stickler syndrome, the exact scoring or questions might need adapting. Further research to ensure other types of Stickler syndrome can be diagnosed early is required. Further validation in other paediatric age ranges is also required, though the lack of change in final score across our age range implies that the tool would retain sensitivity and specificity beyond it.

Looking forwards, our group intends to test this screening tool in a population of paediatric Perthes disease patients, but there are other potential high-risk populations that may benefit from screening for STL1. Early-onset high myopia has been shown to be an area of high genetic yield, with Stickler syndrome predominant, and may be an excellent target for future research [7, 31]. This tool also offers support for diagnosis of STL1 in musculoskeletal, ophthalmology, audiology, cleft palate and general paediatric clinics, where these patients may present to healthcare professionals.

In conclusion, this research has demonstrated the ability of an easy-to-use screening tool to identify children with STL1. Utilization of the tool will allow referral for further ophthalmic and genetic assessment and lead to earlier diagnosis of patients with STL1, giving the vital opportunity for prophylactic cryoretinopexy and prevention of sight loss and blindness in children and their families (Table 5).

**Table 5:** Final screening tool for Stickler syndrome type 1, with questions, scores and threshold for referral for further assessment.

Ophthalmic	Myopia (short-sightedness) before the age of 10 years	2
	Retinal detachment	4
	Family history of retinal detachment: single relative	2
	Family history of retinal detachment: >1 relative	4
Orofacial	History of cleft palate repair	3
	High-arched palate or bifid uvula on examination (if no previous cleft palate repair)	2
	Family history of cleft palate repair	2
Auditory	Sensorineural hearing loss	2
	Family history of deafness	1
Musculoskeletal	Spinal abnormalities (flattened vertebrae, end plate changes, scoliosis, kyphosis)	2
	Family history of atraumatic total hip replacement aged <50 years	2
	Hypermobility on examination (Beighton score ≥6) (If unable to complete Beighton score, elbow hyperextension >10° alone can be assessed for a score of 1).	2
Patients with a final score ≥3 should be further assessed for Stickler syndrome, via referral to local ophthalmology services or the NHS Highly Specialised Service for Stickler syndrome.		

## Supplementary Material

keag389_Supplementary_Data

## Data Availability

The data underlying this article are available in Apollo—University of Cambridge Repository, at https://doi.org/10.25504/FAIRsharing.38c26a.
