# Supplementary material for: A simple questionnaire to prevent blindness: a cross-sectional study screening for Stickler syndrome in children
Source: Rheumatology (Oxford). 2026 Jul 31;65(8):keag389. doi: 10.1093/rheumatology/keag389 (PMC13499231; doi:10.1093/rheumatology/keag389)
Supplement: Supplementary file 1 [file keag389_supplementary_data.zip › rhe-26-0200-File008.docx]

**Supplementary Data S1 – Demographics**

There was no difference in sex between the Stickler and control groups, with a non-significant Chi-squared p-value of 1.000.

| **Group** | **F** | **M** | |
| --- | --- | --- | --- |
| Stickler | 15 | 13 | |
| Control | 13 | 13 | |
| Chi-squared p-value | | | 1.000 |

There was no difference in age between the Stickler and control groups, with a median age of 7 years in both. The p-value of 0.923 was non-significant.

| **Group** | **Median Age (SD)** | **N** |
| --- | --- | --- |
| Stickler | 7 (3.25) | 28 |
| Control | 7 (2.0) | 26 |
| Mann-Whitney U test p-value | | 0.923 |

The distribution of ages can be seen to be similar in each group in the jitter plot below.

Figure – jitter plot of ages of Stickler versus control participants.

Alt text: graphic representation of ages of Stickler and control participants, showing similar distribution of ages in each group.

The ethnicity data broadly showed a predominance to White British, though there were several participants that had not provided data on their ethnicity in both groups. There were also more individuals of other ethnicities than white that were recorded in the control group.

Figure - 15 of the Stickler participants were ‘White British’, whilst 1 was ‘White Irish’. The remaining 12 did not have an ethnicity recorded on their electronic records. The control group similarly had a similar distribution with predominantly ‘White British’ participants (14) alongside ‘White Other’ (2), ‘Asian Bangladeshi’ (1), ‘Asian Indian’ (1), ‘Mixed White and Asian’ (1), ‘Mixed White and Black African’ (1) and 6 listed as blank or other.

Alt Text: Two pie charts showing the distribution of ethnicities in the control and Stickler groups. The majority of participants in both were ‘White British’, and many did not specify an ethnicity.

There was also a spread of Stickler participants from across the UK and Ireland, with no more than two participants represented per county.

Figure – Stickler group participant locations by county across UK and Ireland. One further participant recruited via the SSUK eSurvey lived in the US. All control group participants resided in the catchment of CUH.

Alt Text: Graphic illustration of the location of Stickler participants throughout the UK and Ireland. Most participants are shown as originating from south and mid-England, with some originating from further afield. No one individual area is disproportionately represented in the figure.
