# Supplementary material for: A simple questionnaire to prevent blindness: a cross-sectional study screening for Stickler syndrome in children
Source: Rheumatology (Oxford). 2026 Jul 31;65(8):keag389. doi: 10.1093/rheumatology/keag389 (PMC13499231; doi:10.1093/rheumatology/keag389)
Supplement: Supplementary file 1 [file keag389_supplementary_data.zip › rhe-26-0200-File009.docx]

**Supplementary Figure S1**

Figure - Group-wise linear regression analysis plot of screening tool final score against age. The line of best fit and data points are coloured to match the appropriate group, and the grey-shading either side of each line represents 95% confidence intervals, both of which cross zero and are non-significant (Stickler coefficient of -0.08, 95% CI -0.56 - 0.39, p=0.725 and control coefficient of 0.23, 95% CI -0.07 - 0.53, p=0.130.

Alt Text: graphic representation of the final screening tool scores plotted against age for the Stickler and control groups. The control group is close to zero, whilst the Stickler group scores higher, but neither changes with age according to the confidence bars on either side, which run both up and down.
